# Clinical efficacy of intravesical gemcitabine combined with ubenimex in patients with non-muscle-invasive bladder carcinoma after transurethral resection of bladder tumor

**DOI:** 10.12669/pjms.38.5.4599

**Published:** 2022

**Authors:** Li-jun Shao, Hai-jiang Wang, Jia-rong Wang, Xiao-fei Yuan, Quan Sha

**Affiliations:** 1 Li-jun Shao, Department of Urology, Baoding No.1 Hospital, Baoding, 071000, Hebei, P.R. China; 2 Hai-jiang Wang, Department of Urology, Baoding No.1 Hospital, Baoding, 071000, Hebei, P.R. China; 3 Jia-rong Wang Department of Oncology, Baoding Children’s Hospital, Baoding, Hebei, 071051, P.R. China; 4 Xiao-fei Yuan, Department of Urology, Baoding No.1 Hospital, Baoding, 071000, Hebei, P.R. China; 5 Quan Sha, Department of Urology, Baoding No.1 Hospital, Baoding, 071000, Hebei, P.R. China

**Keywords:** Gemcitabine, Immunotherapy, Urothelial carcinoma of the bladder, Non-muscle-invasive, Transurethral resection of bladder tumor

## Abstract

**Objectives:**

To evaluate the clinical value of intravesical gemcitabine combined with immunotherapy in patients with non-muscle-invasive bladder carcinoma (NMIBC) after transurethral resection of bladder tumor (TURBT).

**Methods:**

Eighty patients with non-muscle-invasive urothelial carcinoma treated in Baoding No.1 Hospital from November 2016 to November 2019 were randomly divided into two groups, with 40 patients in each group. Both groups underwent TURBT. After surgery, the research group was treated with intravesical chemotherapy using gemcitabine combined with ubenimex, while the control group was given 40 mg pirarubicin by intravesical instillation. Postoperative condition was evaluated by cystoscopy every three months in both groups. The recurrence six months, one year and two years after treatment, the incidence of lower urinary tract symptoms such as dysuria, hematuria and frequent urination, general adverse drug reactions such as rashes, liver function damage and gastrointestinal reaction, as well as the changes in CD3^+^, CD4^+^, CD8^+^ and CD4^+^/CD8^+^ T lymphocyte subsets before and after treatment were comparatively analyzed between the two groups.

**Results:**

The recurrence rate showed no statistical significance between the two groups 6 months after treatment (*p*=0.17), but significant differences one year (*p*=0.04) and two years (*p*=0.03) after treatment, which were significantly lower in the research group than the control group. The incidence of adverse drug reactions was 22.5% in the research group and 7.5% in the control group, without significant difference (*p*=0.36). The incidence of lower urinary tract symptoms was 32.5% and 55%, respectively, in the research group and the control group. The incidence of lower urinary tract symptoms in the research group was significantly lower compared with the control group, with a statistically significant difference (*p*=0.04). After treatment, CD3^+^, CD4^+^ and CD4^+^/CD8^+^ levels in the research group increased significantly than those in the control group, with statistically significant differences (CD3^+^, *p*=0.01; CD4^+^, *p*=0.00; CD4^+^/CD8^+^, *p*=0.00).

**Conclusions:**

For NMIBC patients receiving bladder-preserving surgery, intravesical gemcitabine combined with immunotherapy can reduce the recurrence rate, relieve lower urinary tract symptoms, increase the tolerance of patients to intravesical chemotherapy and significantly improve the function of T lymphocytes, without obvious increase in adverse drug reactions. Therefore, it is safe and effective, and has certain clinical value.

## INTRODUCTION

Bladder carcinoma is a malignant tumor originating from the bladder urothelium, which is a common malignant tumor of the urinary system, with higher incidence in males than females.^1^ With the improvement of people’s living standard, its incidence is increasing year by year.^2^ Most of the patients suffer from non-muscle-invasive bladder carcinoma (NMIBC), accounting for 75-85% of the patients with new bladder carcinoma.^3^ At present, for patients without surgical contraindications, cystoscopy and transurethral resection of bladder tumor (TURBT) are still the gold standard for NMIBC treatment because of their minimal invasion, less pain and rapid postoperative recovery.^4^ However, NMIBC still has a high risk of recurrence after surgery, and postoperative adjuvant intravesical chemotherapy is needed to eliminate residual tumor cells and reduce the recurrence rate.^5^ Currently, there are many drugs used in intravesical chemotherapy, such as mitomycin, pirarubicin and epirubicin, which have no obvious advantage in preventing recurrence compared with another drug.^6^ As a novel type of anti-tumor drug, gemcitabine resist tumors mainly through inhibiting tumor cells at the DNA synthesis phase, thus blocking the proliferation of tumor cells, and has shown obvious anti-bladder tumor activity and low toxicity.^7^ In this study, gemcitabine combined with ubenimex was used, confirming that this regime used in intravesical chemotherapy after TURBT could reduce the recurrence rate, alleviate lower urinary tract symptoms, increase the tolerance of patients to intravesical chemotherapy, and significantly improve T-lymphocyte function, with certain clinical value.

Our objective was to evaluate the clinical value of intravesical gemcitabine combined with immunotherapy in patients with non-muscle-invasive bladder carcinoma (NMIBC) after transurethral resection of bladder tumor.

## METHODS

Eighty patients with NMIBC treated in Baoding No.1 Hospital from November 2016 to November 2019 were selected and randomly divided into two groups, with 40 patients in each group. In the research group, there were 23 males and 17 females, aging 43-71 years (average age, 60.74 ± 8.14 years). The control group included 25 males and 15 females, aging from 38 to 73 years, with an average age of 61.08 ± 8.13 years. No significant differences were found in general data between the two groups, suggesting comparability (Table-I).

**Table I:** Comparative analysis of general data between research group and control group (χ¯±S), n = 40).

Indicator	Research group	Control group	t/χ^2^	*p*
Age (years)	60.74 ± 8.14	61.08 ± 8.03	0.19	0.85
Male (n, %)	23 (57.5%)	25 (62.5%)	0.21	0.65
Number of tumors (n)	1.64 ± 0.78	1.59 ± 0.92	0.26	0.79
Tumor diameter (cm)	1.37 ± 0.12	1.40 ± 0.23	0.73	0.47

*P* > 0.05.

### Ethical Approval:

The study was approved by the Institutional Ethics Committee of Baoding Children’s Hospital (No.: 1951ZF042; date: June 18, 2018), and written informed consent was obtained from all participants.

### Inclusion criteria:

- Patients with bladder carcinoma suggested by imaging examinations such as CT^8^;
- Patients with pathologically confirmed low-grade urothelial carcinoma^9^;
- Patients aged 30-75 years;
- Patients with indications for surgical treatment and postoperative pathological results;
- Patients’ family members with willingness and ability to cooperate with this study, and patients with high treatment compliance;
- Patients with complete clinical data and imaging data;
- Patients with tumors ≤ 3.

### Exclusion Criteria:

- Patients complicated with severe underlying diseases, and intolerant to surgery;
- Patients complicated with malignant tumors in other parts;
- Patients accompanied by urinary tract infection and other factors affecting the results of this study;
- Patients with mental disorders, and inability to cooperate with this study;
- Patients with incomplete clinical data;
- Patients orally administrated with relevant drugs affecting this study recently;
- Patients with high-grade tumors and myometrial invasion suggested by postoperative pathological results.

The patients in both groups were treated with TURBT. Under general anesthesia, the patients were in the lithotomy position, with routine disinfection, towel laying and connection to light source, camera and flusher. The urethra of male patients was disinfected with diluted iodophor solution to prevent retrograde infection. A cystoscope was inserted into the bladder to determine the location, number and size of the lesions, with clear visual field maintained by continuous irrigation with normal saline. The tumor was enucleated to the muscle layer within 2 cm around the tumor using the green laser system, and the blood vessels and bleeding points at the basal part and around the tumor were cauterized. After complete hemostasis, the cystoscope was withdrawn, and the bladder was continuously flushed with sterilized distilled water for 24 hour..

The research group was treated with intravesical chemotherapy using gemcitabine combined with ubenimex. Intravesical instillation was performed using 1.2 g gemcitabine once a week for the first eight times and once a month later for one year. Gemcitabine Tablets (30 mg) were administrated three times/daily for three months. The control group was given 40 mg pirarubicin by intravesical instillation, once a week for the first eight times and once a month later, a total of one year. Postoperative condition was evaluated by cystoscopy every threemonths in both groups.

### Observation indicators:

Evaluation of recurrence rate: The recurrence six months, one year and two years after surgery of all the patients were recorded, to comparatively analyze the difference in recurrence rate between the two groups. The incidences of general adverse drug reactions including rashes, liver function damage and gastrointestinal reaction were compared and analyzed between the two groups. The incidences of lower urinary tract symptoms such as dysuria, hematuria, frequent urination and cystitis were compared and analyzed between the two groups. Analysis of immune status: Fasting blood samples were taken in the morning before and after treatment for detecting the levels of CD3^+^, CD4^+^, CD8^+^ and CD4^+^/CD8^+^ T lymphocyte subsets, and their differences were compared and analyzed between the two groups before and after treatment.

### Statistical Analysis:

All data were statistically analyzed using SPSS 20.0. The measurement data were expressed as (X̄ ± S). Inter-group analysis was carried out using the two independent samples t-test, intra-group analysis by the paired t-test, and the comparison in percentage with the χ^2^ test. *P*< 0.05 was considered as statistically significant.

## RESULTS

The comparative analysis of recurrence rate between the two groups after treatment is shown in Table-II. The recurrence rate showed no statistically statistical significance between the two groups six months after treatment (*p*=0.17), but significant differences one year (*p*=0.04) and two years (*p*=0.03) after treatment, which were significantly lower in the research group than the control group (Table-II).

**Table II:** Comparative analysis of recurrence rate between two groups after treatment (χ¯ ± S, n = 40).

Group	6 months after surgery	1 year after surgery *	2 years after surgery *
Research group	1 (2.5%)	2 (5%)	4 (10%)
Control group	4 (10%)	8 (20%)	11 (27.5%)
χ^2^	1.92	4.11	4.02
*p*	0.17	0.04	0.03

* *p*< 0.05.

*Comparative analysis of incidences of general adverse drug reactions between two groups* Comparative analysis after treatment revealed that the incidence of adverse drug reactions was 22.5% in the research group and 7.5% in the control group, without significant difference (*p*= 0.36), as seen in Table-III.

**Table III:** Comparative analysis of general adverse drug reactions between two groups (χ¯±S, n = 40).

Group	Rashes	Gastrointestinal reaction	Edema	Liver function damage	Incidence
Research group	2	3	1	3	9 (22.5%)
Control group	2	0	0	1	3 (7.5%)
χ^2^					3.53
*p*					0.06

*p*> 0.05.

After treatment, the incidence of lower urinary tract symptoms was 32.5% and 55%, respectively, in the research group and the control group. The incidence of lower urinary tract symptoms in the research group was significantly lower compared with the control group, with a statistically significant difference (*p*=0.04), as displayed in Table-IV.

**Table IV:** Comparative analysis of lower urinary tract symptoms between two groups after treatment (χ¯±S, n = 40).

Group	Hematuria	Dysuria	Frequent urination	Cystitis	Total
Research group	2	4	4	3	13 (32.5%)
Control group	3	8	7	4	22 (55%)
χ^2^					4.11
*p*					0.04

*p*< 0.05.

Before treatment, CD3^+^, CD4^+^ and CD4^+^/CD8^+^ levels presented no significant differences between the two groups (*p*>0.05). After treatment, CD3^+^, CD4^+^ and CD4^+^/CD8^+^ levels in the research group increased significantly than those in the control group, with statistically significant differences (CD3^+^, *p*=0.01; CD4^+^, *p*=0.00; CD4^+^/CD8^+^, *p*=0.00). However, CD8^+^ did not change significantly (*p*= 0.91) (Table-V).

**Table V:** Comparison in T lymphocyte subsets between two groups before and after treatment (χ¯±S, n = 30).

Indicator		Research group Δ	Control group Δ	t	*p*
CD3+(%)	Before treatment *	42.95 ± 6.43	43.68 ± 7.06	0.48	0.63
	After treatment Δ	50.07 ± 6.38	46.89 ± 7.15	2.81	0.01
	t	4.97	2.08		
	*p*	0.00	0.04		
CD4+(%)	Before treatment *	25.72 ± 4.37	26.47 ± 5.06	0.71	0.48
	After treatment Δ	38.48 ± 5.20	33.54 ± 5.42	4.16	0.00
	t	11.88	6.03		
	*p*	0.00	0.00		
CD8+(%)	Before treatment *	22.57 ± 5.86	22.64 ± 3.78	0.06	0.94
	After treatment *	22.85 ± 5.38	22.73 ± 3.91	0.11	0.91
	t	0.22	0.10		
	*p*	0.82	0.92		
CD4+/CD8+	Before treatment *	1.35 ± 0.44	1.37 ± 0.36	0.22	0.82
	After treatment Δ	1.92 ± 0.57	1.58 ± 0.49	2.98	0.00
	t	5.01	2.34		
	*p*	0.00	0.02		

* *p*> 0.05, Δ*p*< 0.05.

## DISCUSSION

Clinically, bladder carcinoma is one of the most common urogenital cancers in the Department of Urology, characterized by high incidence and high recurrence rate.^10^ Its main risk factors include smoking, exposure to certain chemicals in work and general environment and some genetic factors, which all have a certain impact on this disease.^11^ With the continuous progress of diagnosis and treatment technology and equipment, most patients (70-80%) are early non-muscle-invasive when diagnosed.^12^ The aim of NMIBC treatment is to clear the primary lesion and reduce the chance of tumor recurrence or progression.^13^ At present, TURBT is still the main treatment for NMIBC, but there is a risk of residual tumor and recurrence after surgery.^14^

Intravesical instillation of chemotherapeutic drugs can kill the tumor cells that disseminated during transurethral resection of tumor, as well as the residual tumor cells at the cutting edge and the tumor tissues that can not be distinguished by naked eyes. Therefore, intravesical chemotherapy after TURBT is an effective measure to reduce the recurrence rate.^15^ However, due to high heterogeneity, there are some differences in the choice of chemotherapeutic drugs and the results of tumor control. Consequently, in the selection of clinical drugs, appropriate chemotherapeutic drugs should be selected according to the disease development characteristics of patients.^16^ At present, dozens of chemotherapeutic drugs are used for intravesical instillation, but their efficacy is not marked.^17^

The ideal chemotherapeutic drugs for intravesical instillation should have high specificity for bladder tumor cells and high local drug concentration, with less systemic absorption and mild adverse reactions, and can also effectively prevent or reduce the recurrence rate of tumors. A novel cytosine nucleoside derivative, gemcitabin is activated by deoxycytosine kinase and metabolized by cytidine deaminase after entering the human body. Gemcitabine is a pyrimidine anti-tumor drug, whose main metabolites are incorporated into DNA in cells, which mainly act at the G1/S phase,^18^ and can inhibit deoxycytidine deaminase and reduce the degradation of intracellular metabolites, with a self-synergistic effect. In clinic, gemcitabine is effective for multiple solid tumors.^19^ In a multicenter, randomized, prospective trial,^20^ the efficacy of intravesical instillation using gemcitabine was evaluated, suggesting that gemcitabine might be the second-line treatment for high-risk NMIBC patients after BCG failure. Additionally, Álvarez M et al.^21^ believed that taxane and gemcitabine alone or their combination had obvious advantages for postoperative recurrence of bladder tumor after bladder-preserving surgery, and could reduce the chance of tumor recurrence. Moreover, Milbar et al.^22^ Considered that bladder-preserving surgery rather than radical cystectomy was the first choice for recurrent NMIBC, and postoperative gemcitabine instillation had obvious therapeutic advantages, without severe adverse reactions. Further, Steinberg et al.^23^ Compared the efficacy and safety of intravesical chemotherapy with gemcitabine and pirarubicin in the prevention of recurrence in patients with NMIBC after TURBT, and found that the recurrence-free survival time of patients receiving gemcitabine chemotherapy was longer than that of those treated with pirarubicin, and the adverse reactions were milder. In our study, it was also confirmed that the recurrence rate was not statistically significant between the two groups 6 months after treatment (*p*=0.17), but significantly different 1 year (*p*=0.04) and 2 years (*p*=0.03) after treatment, which were significantly lower in the research group than the control group. In addition, the incidence of lower urinary tract symptoms was 32.5% and 55%, respectively, in the research group and the control group. The incidence of lower urinary tract symptoms in the research group was significantly lower compared with the control group, with a statistically significant difference (*p*= 0.04).

The immune function of the body is closely related to the occurrence and development of tumors. A study^24^ has shown that the recurrence and metastasis of bladder carcinoma are closely related to the expression of aminopeptidase N (APN), which belongs to the aminopeptidase family and is widely distributed in all animals and plants. APN is considered to be a very important target of cancer treatment in that it is related to the progression and metastasis of cancers^25^. Ubenimex can be used as an APN inhibitor to inhibit the degradation of extracellular matrix during tumorigenesis. Additionally, ubenimex can also induce the death and apoptosis of autophagocytes, which indicates that a mixed type of programmed cell death occurs in bladder tumor cells after ubenimex application.^26^ Toshiyama et al.^27^ Found that ubenimex and fluorouracil, cisplatin or adriamycin had a synergistic effect and played an effective anti-tumor role, without increase in adverse drug reactions. Moreover, ubenimex is also an inhibitor of CD13, which can be used as an immune adjuvant to improve the immune status of the body.^28^ According to the meta-analysis of Hu et al.^29^ Ubenimex is widely used as an immunoregulator in the treatment of leukemia, non-small-cell lung cancer and bladder carcinoma to improve the effect of anti-tumor therapy. It has a positive effect on the improvement of cellular immune state in the body.^30^ Our study also proved that after treatment, CD3^+^, CD4^+^ and CD4^+^/CD8^+^ levels in the research group increased significantly than those in the control group, with statistical significance (CD3^+^, *P* = 0.01; CD4^+^, *P* = 0.00; CD4^+^/CD8^+^, *P* = 0.00), indicating that the immune status of T lymphocytes in patients treated with ubenimex was improved.

### Limitations of this study:

The sample size is small, bladder tumor is a disease with high recurrence, and the follow-up time is not long enough. Additionally, considering the safety of patients, only pirarubicin was included in the study and compared with this regime, but the comparative analysis of other chemotherapeutic drugs for intravesical instillation was not involved. We are also further enlarging the sample size, as well as increasing the comparative study of other chemotherapeutic drugs, and the effect analysis of intravesical instillation using gemcitabine at different doses combined with ubenimex, in the expectation of more comprehensive evaluation of the treatment benefits of NMIBC patients after bladder-preserving surgery.

## CONCLUSION

In conclusion, for NMIBC patients receiving bladder-preserving surgery, intravesical gemcitabine combined with immunotherapy can reduce the recurrence rate, relieve lower urinary tract symptoms, increase the tolerance of patients to intravesical chemotherapy and significantly improve the function of T lymphocytes, without obvious increase in adverse drug reactions. Therefore, it is safe and effective, and has certain clinical value.

### Authors’ Contributions:

**LJS &** **HJW:** Designed this study and prepared this manuscript, and are responsible and accountable for the accuracy or integrity of the work.

**JRW:** Collected and analyzed clinical data.

**XFY & QS:** Significantly revised this manuscript.
